# Immunologic and Non-Immunologic Mechanisms Leading to Airway Remodeling in Asthma

**DOI:** 10.3390/ijms21030757

**Published:** 2020-01-23

**Authors:** Lei Fang, Qinzhu Sun, Michael Roth

**Affiliations:** 1 Pulmonary Cell Research & Pneumology, University Hospital & University of Basel, Petersgraben 4, CH-4031 Basel, Switzerland; Lei.fang@unibas.ch; 2 College of Animal Science and Technology, Northwest A&F University, Yangling 712100, Shaanxi, China; sunqingzhu@nwafu.edu.cn

**Keywords:** asthma, airway wall remodeling, regulatory mechanisms

## Abstract

Asthma increases worldwide without any definite reason and patient numbers double every 10 years. Drugs used for asthma therapy relax the muscles and reduce inflammation, but none of them inhibited airway wall remodeling in clinical studies. Airway wall remodeling can either be induced through pro-inflammatory cytokines released by immune cells, or direct binding of IgE to smooth muscle cells, or non-immunological stimuli. Increasing evidence suggests that airway wall remodeling is initiated early in life by epigenetic events that lead to cell type specific pathologies, and modulate the interaction between epithelial and sub-epithelial cells. Animal models are only available for remodeling in allergic asthma, but none for non-allergic asthma. In human asthma, the mechanisms leading to airway wall remodeling are not well understood. In order to improve the understanding of this asthma pathology, the definition of “remodeling” needs to be better specified as it summarizes a wide range of tissue structural changes. Second, it needs to be assessed if specific remodeling patterns occur in specific asthma pheno- or endo-types. Third, the interaction of the immune cells with tissue forming cells needs to be assessed in both directions; e.g., do immune cells always stimulate tissue cells or are inflamed tissue cells calling immune cells to the rescue? This review aims to provide an overview on immunologic and non-immunologic mechanisms controlling airway wall remodeling in asthma.

## 1. Introduction

The prevalence of asthma has been increasing worldwide for at least three decades, without any definite reason. Recent evidence suggested that this increase of asthma cases might be linked to urban air pollution, with the fact that more and more people are attracted to living in cities [1,2,3,4,5]. According to the WHO, 30% of all child deaths before the age of 5 were due to asthma caused by polluted air (http://www.who.int/mediacentre/news/releases/2017/pollution-child-death/en/). These arguments were supported by the report on the “State of Global Air 2017” (https://www.healtheffects.org/ announcements/hei-launches-state-global-air-report-and-website). The European Union acknowledged that, “chronic airway diseases are a major and growing health problem in Europe” [6]. The contribution of asthma to health care costs, as well as costs to the patient’s life are largely underestimated. As an example in the USA, the costs of asthma care exceeded that of HIV and tuberculosis combined in 2015 [7].

Air pollution can stimulate the expression of pro-inflammatory cytokines in the lung, not only by immune cells, but also by tissue forming cells [8,9]. Despite initiating inflammation, the inflammatory effect of air pollutants may be further increased in people with existing allergies or when appearing together with allergens [10,11,12]. The mechanism(s) by which air pollution alters the structure of the lung tissues is not well understood, but it was suggested that the interaction between the environment and epigenetic events plays a major role in the pathogenesis of asthma [13]. Thus, the mechanisms by which air pollution might trigger asthma can be activated by immunological and non-immunological responses of the lung.

In recent years, the search for the initiating event that starts the pathogenesis of asthma was extended to the analysis of the genome, transcriptome, proteome, secretome, inflammasome, metabolome, and microbiome, which did not deliver the expected results. As reviewed by Tyler and Bunyavanich [14], the application of different “-omic” analyses did not identify new biomarkers for specific asthma phenotypes or endotypes. There was also no study that provided evidence for mechanisms which could explain the origin of airway wall remodeling in asthma [14]. Most studies confirmed already known biomarkers for asthma, but did not indicate any new targets for diagnosis or therapy. The only new aspect that seems to show up in many studies was the important role of the interaction between the environment and the lung, based on epigenetic gene regulation. Many of the “-omic” studies indicated that tissue remodeling is a major pathology and against previous assumption occurs independent from inflammation [15,16,17]. Furthermore, many studies reported that the different asthma phenotypes, which were described based on patient symptoms, could not be well separated by specific cellular pathologies [18,19,20]. These findings suggest that the pathogenesis of asthma must be re-evaluated, by not focusing solely on the immune response.

## 2. Asthma

The definition of asthma phenotypes is not standardized and is either based on the patients’ history and the clinical symptoms, or on the presence of type-1 (neutrophilic-) and type-2 (eosinophilic-) cytokines [21]. Others classified asthma according to the triggers as allergic asthma, non-allergic asthma, adult onset asthma, or childhood asthma. However, each of these categories could further be subdivided according to the profile of type-1 or type-2 cytokines [22]. In this study, it was hypothesized that type-2 cytokines play no role during the initiation phase of asthma. Instead, it was suggested that the earliest events depend on the interaction of bronchial epithelial cells with various environmental factors, including viruses and microorganisms.

**Figure 1:**
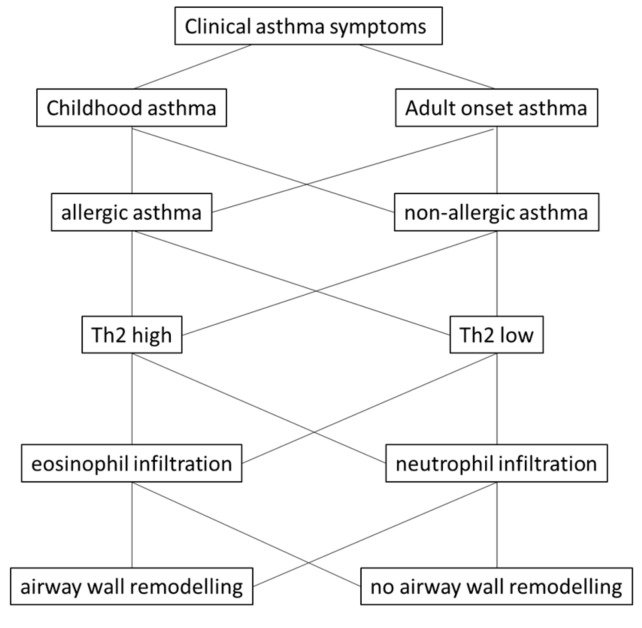
The most frequently used asthma categories and how they can be subdivided based on the history and on symptoms. Asthma patients can be classified as “childhood” or “adult”-onset asthma. These two categories can further be classified by allergic tests as “allergic” or “non-allergic” asthma. For further classification cytokine expression assays can be performed in blood or sputum based indicating patients as type-2 “high” or “low”. These two categories often correlate with the presence of eosinophils or neutrophils, respectively. However, airway wall remodeling occurs in most asthma patients and does not correlate with specific pheno- or endo-types [15,16].

However, there is no clear hypothesis regarding by which mechanism(s) the microorganisms and non-biological factors, such as dust, lead to the same clinical symptoms that are diagnosed as asthma. Nevertheless, the hypothesis is supported by the observation that airway wall remodeling often occurs prior to the detection of any inflammation [16,23]. In children with severe asthma, airway wall remodeling correlated with eosinophilia, but not with type-2 cytokines [24]. The link between inflammation and airway wall remodeling in childhood asthma was analyzed by Castro-Rodriguez et al. [17], performing a meta-analysis over 39 studies including 2390 children under the age of 18 years. This study suggested that airway inflammation and remodeling was not present in children under 12 months of age, but occurred in older children. The major changes in the tissue structure were an increase of the basement membrane thickness, and of airway smooth muscle cells. The study concluded that the failure to prove eosinophilic inflammation in children without airway wall remodeling rejected the hypothesis that inflammation is the cause of airway wall remodeling. This is in line with the observation that in adult asthma, airway wall remodeling was independent of inflammation and occurred after repeated challenge of volunteers with allergens or methacholine [25]. The study concluded that airway wall remodeling results from frequent constriction of the tissue, rather from inflammation. Figure 1 provides an overview of asthma classification and sub-classes.

There is surprisingly little evidence in human that type-2 cytokines can cause airway wall remodeling. So far, only the increased expression of IL-13 was linked to epithelial dysfunction in humans [26]. Indirect evidence suggested that inhibition of IL-13 did not affect airway wall remodeling, but reduced eosinophilic inflammation in asthma [27]. However, a new study suggested, that IL-13 might be essential for the maintenance of the airway epithelium and regulates wound repair [28]. If this function of IL-13 is confirmed in humans, the long-term application of neutralizing IL-13 therapies might cause more damage to the airways than it helps to control asthma. A similar conclusion can be made for neutralizing IL-4, which is not surprising since both interleukins IL-4 and IL-13 signal through the same receptor complex. In addition, most studies that investigated the role of type-2 and type-17 cytokines on airway wall remodeling were performed in animal models of allergic asthma, or in isolated human cell lines [29,30]. It should also be considered that airway wall remodeling caused the increased expression of type-2-cytokines by human airway epithelial cells [31]. These findings indicate that bi-directional crosstalk between tissue forming cells and immune cells exists, and plays an important role in the pathogenesis of asthma.

## 3. Immunologic and Non-Immunologic Stimulation of Airway Wall Remodeling in Asthma

Airway wall remodeling was recognized as an independent and important pathology in asthma that describes structural changes of the airway wall tissue forming cells, as well as changes of the composition of its extracellular matrix. The term “remodeling” is not well defined, it includes the disruption of the epithelium [32], or the accumulation of extracellular matrix in the fibroblast layer [33], or hyperplasia/hypertrophy or airway smooth muscles [34,35,36], or reduced apoptosis of airway wall cells [37]. Any of these pathologies can occur alone or in combination during the course of asthma. Only few studies investigated if there is a difference in the pattern of airway wall remodeling comparing allergic to non-allergic asthma. All studies reported that the structural changes are more severe in allergic asthma, but none found significant differences compared to non-allergic asthma [38,39].

An increase of the airway smooth muscle mass was the first tissue pathology that was associated with severe and fatal asthma [40]. The importance of the airway smooth muscle cells to airway wall remodeling has been confirmed by subsequent studies [41,42,43]. Interestingly, epigenetic events occurring in a cell type specific manner in airway smooth muscle cells were identified as the driving force behind remodeling and inflammation [43].

In the context of allergic asthma, it is of interest that the contribution of IgE to remodeling of the airways may occur directly on airway smooth muscle cells, which express the high and low affinity receptor for IgE [44]. Moreover, airway smooth muscle cells could be activated by the presence of IgE alone in the absence of allergens [45,46,47,48,49]. In vitro, remodeling of airway smooth muscle cells and inflammation occurred through different signaling pathways [47,48]. Proliferation and secretion of pro-inflammatory cytokines was induced by non-allergen IgE in human airway smooth muscle cells by activating mitogen activated protein kinases (MAPK) and STAT3 [46,47,48]. Furthermore, proliferation of airway smooth muscle cells by IgE was independent of allergens and inhibited by IgE neutralizing antibodies [49]. IgE stimulated the differentiation and increased the constriction of airway smooth muscle cells [50]. Furthermore, IgE-receptor I activated phosphoinositid-3 kinase (IP3K), leading to the signaling through the Akt → mTOR → p70S6 kinase → peroxisome proliferator-activated receptor (PPAR)-γ and its co-activator PGC-1α, therefore influence mitochondrial function to support airway remodeling. This signaling cascade can be blocked by the Akt inhibiting protein phosphatase and tensin homolog (PTEN), a mechanism that is reduced by IgE in asthmatic airway cells [51].

**Figure 2:**
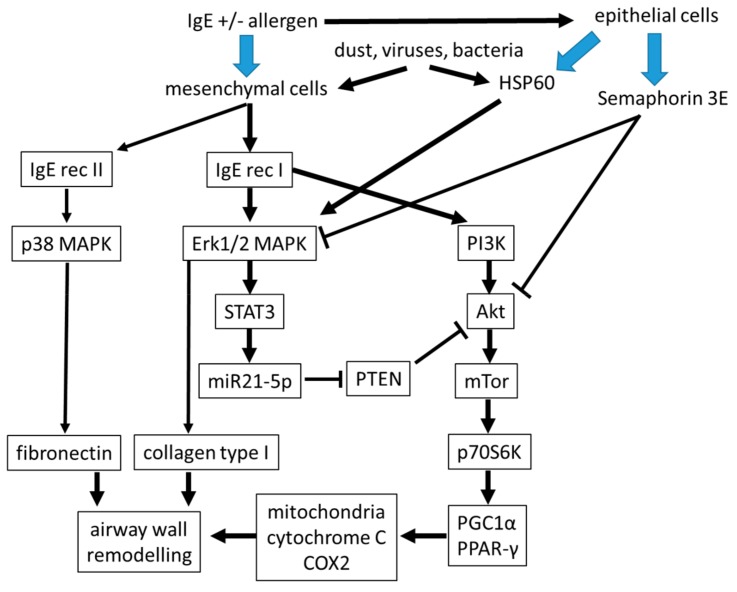
The suggested link intracellular signaling in IgE-stimulated airway mesenchymal cells. The function of sub-epithelial mesenchymal cells is a major factor for tissue homeostasis of the airway wall. It is indicated that their function can either be modified by direct binding of IgE to mesenchymal cells, or indirectly by mediators released by epithelial cells. MAPK: mitogen activated protein kinase, PI3K: phospho-inostitol-3 kinase, HSP60: heat shock protein-60, PTEN: Phosphatase and Tensin homolog, STAT3: signal transducer and activator of transcription 3, miR: microRNA, Akt: serine/threonine kinase Akt, also known as protein kinase B (PKB), p70S6K: protein70-S6-kinase, mTor: mammalian target of rapamycin, PGC1α: Peroxisome proliferator-activated receptor-gamma coactivator (PGC)-1alpha, PPAR-γ: Peroxisome proliferator-activated receptor-gamma.

The action of IgE might be blocked by semaphorin 3E expression that was reduced in cells isolated from patients with severe allergic asthma [52]. However, clinical proof for the reducing action of anti-IgE antibodies on airway wall remodeling is missing. Semaphorin 3E was implied to reduce remodeling of airway smooth muscle cells and angiogenesis induced by house dust mite exposure in an animal model [53,54]. Overexpression of semaphorin 3E, or intranasal administration in mice, significantly reduced eosinophilic inflammation, serum IgE, and type-2-cytokine expression [55]. This makes semaphoring 3E an interesting candidate for the diagnosis and therapy of asthma, but its role in the pathogenesis of airway wall remodeling needs to be further investigated (Figure 2).

Several cell type specific molecular pathologies have been described in asthmatic airway smooth muscle cells including increased mitochondria and Erk1/2 MAPK expression, and low cAMP levels [36,55,56]. These cell type specific pathologies might contribute to the activation status of airway wall mesenchymal cells as shown in Figure 2. In addition, the composition of the extracellular matrix within the sub-epithelial cell layers was modified in asthma and maintained in isolated fibroblasts and smooth muscle cells of asthma patients [33,57]. Together, these factors caused the increased capacity of smooth muscle cells to proliferate, which was reported earlier [58,59,60].

The observation that the extracellular matrix obtained from mesenchymal airway wall cells of asthma patients increased the production of pro-inflammatory type-2-cytokines [31], suggest a pro-inflammatory feedback mechanism between tissue forming airway wall cells and the immune system. Therefore, the role of the extracellular matrix composition and its contribution to the pathogenesis of asthma has to be studied in more detail. As reviewed by Boulet [60], the increased proliferation of smooth muscle cells in asthma is not responsive to any available drug or biological therapy; only bronchial thermoplasty lastingly reduced smooth muscle mass in patients with severe asthma. Thus, several of these pathologies should be considered in the search for future targets in asthma therapy and diagnosis [61].

Moreover, the above-mentioned intracellular signaling pathways can be activated by asthma relevant micro-organisms such as rhinovirus, respiratory syncytial virus (RSV), bacteria, or intracellular parasites [62,63,64,65,66]. However, we are just starting to understand the mechanisms by which these different micro-organisms activate intracellular signaling of host cells and how they use this for their own benefit. Many asthma relevant micro-organisms induce the production of pro-inflammatory cytokine including IL-4, IL-13, and TGF-β, which are well known as contributors to the pathogenesis of asthma. The fact that micro-organisms activate the same signaling pathways and cytokines as other asthma triggers might explain how they stimulate airway wall remodeling [62,63]. Dependent on the host cell type, there is also evidence that for example RSV directly affect the interaction between mesenchymal cells and epithelial cells [65], and lead to airway hyper-reactivity [66]. The interaction between the tissue forming cells, also called the epithelial-mesenchymal trophic unit, is a key mechanism to understand the origin of airway wall remodeling.

In a recent publication, it was suggested that inhibiting prostaglandin D2 receptors reduced airway smooth muscle cell mass in asthma patients [67]. The role of prostaglandin D2, released by mast cells, in the course of asthma exacerbation, was so far seen as a stimulator of eosinophils [68]. There was no indication that airway smooth muscle cells respond to prostaglandin D2 directly. If the model presented by Saunders et al. [67] can be proven, it will open a novel aspect for asthma therapy, especially as an inhibitor of airway wall remodeling.

## 4. Controlling the Epithelial-Mesenchymal Interaction by Immunologic and Non-Immunologic Factors

The epithelial-mesenchymal trophic unit (EMTU), describes the interaction of the epithelium with sub-epithelial mesenchymal cells. This interaction between the two cell layers of the airway wall was regarded as a major regulator of the homeostasis of the airway wall structure, and was described over two decades ago [69,70]. The importance of the EMTU for the maintenance and function of the airway came back into focus recently [71,72]. However, most studies that investigated the interaction between the epithelial cells and sub-epithelial mesenchymal cells focused on a one-way direction from epithelial cells to fibroblasts. Allergens inhaled by volunteering asthma patients triggered the release of alarmins including IL-25, IL-33, and TSLP, which modified the function of the mesenchymal cells in the sub-epithelial cell layer [73]. This observation may further explain the effect of allergens and methacholine on the remodeling, which has been described earlier in patients with very mild asthma [25]. The decreased secretion of folistatin-like 3 protein by airway epithelium impaired the transition of fibroblasts to myo-fibroblasts [74]. The release of basic fibroblast growth factor (bFGF) from epithelial cells was triggered by rhinovirus infection, thereby stimulating sub-epithelial fibroblast remodeling [75]. Exposure of epithelial cells to ozone stimulated collagen synthesis and proliferation of fibroblasts in a co-culture system [76]. In a rhesus monkey model, ozone exposure during early childhood altered the development and maturation of the lung in a very strict pattern, which was controlled through the timing of the damage [77]. A similar effect had been reported earlier in the same animal model for the presence of CD25 cells after an allergen challenge [78]. These changes of the lung structure may be linked to the modified function of bronchial epithelial cells by environmental factors, and their response to stress. These pathological changes may be explained by our observation that epithelial cells of asthma patients secreted stress proteins such as heat shock protein (HSP)-60, which stimulated fibroblast proliferation and collagen deposition [79].

The effect of fibroblasts on epithelial cells is less well studied. Fibroblasts promoted epithelial cell proliferation by stimulating the transforming growth factor (TGF)-β2 signaling pathway in severe asthma [80]. A similar stimulating effect of fibroblasts on bronchial epithelial cells was reported in horses [71]. In contrast, the secretion of Wnt-5a/b by mesenchymal cells repressed the function of alveolar epithelial progenitor cells, and may therefore indicate a novel strategy to control lung remodeling in chronic inflammatory diseases [81]. TGF-β1 stimulated the expression of the epi-genetic acting protein arginine methyltransferase 1 (PRMT1), which is controlled by several asthma relevant stimuli including HSP60 [79]. The possible role of PRMT1 in the pathogenesis of asthma is described in more detail below.

**Figure 3:**
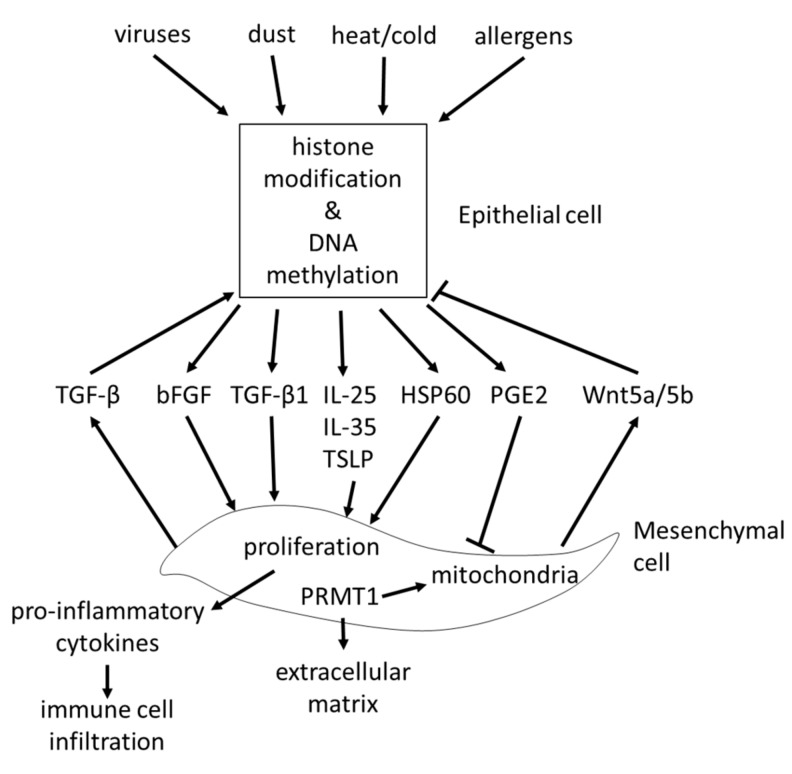
Remodeling controls the interaction between airway epithelial and mesenchymal cells. Different environmental conditions such as heat, dust, viruses, or allergens are suggested to induce similar epigenetic events in epithelial cells. Upon these epigenetic events, epithelial cells release a wide range of pro-inflammatory and remodeling stimulating proteins, which act on sub-epithelial mesenchymal cells and change their function. TGF: transforming growth factor, bFGF: basic fibroblast growth factor, HSP: heat shock protein, IL: interleukin, PGE2: prostaglandin E2, Wnt: wingless, TSLP: thymic stromal lymphopoietin, PRMT1: protein arginine methyltransferase 1.

The interaction between epithelial cells and sub-epithelial mesenchymal cells is summarized in Figure 3.

## 5. Mechanisms to Explain How Air Pollution Triggers Remodeling in Asthma

Air pollution, especially with fine dust, is an increasing global health problem in expanding urban areas, and it was linked to the development of chronic inflammatory lung diseases including asthma [82,83,84]. Fine dusts with a particulate matter size < 2.5 micrometers (PM2.5), can originate from traffic, industry, or agriculture. Independent from its origin, exposure to PM2.5 was linked to systemic inflammation and the increasing prevalence of chronic inflammatory lung diseases [85,86]. Surprisingly, the duration of exposure to PM2.5 was less important. Inhaled PM2.5 accelerated the decline of lung function, caused irreversible airway wall remodeling, and increased the exacerbation rate in asthma patients [87,88]. Even in the healthy lung, PM2.5 correlated with increased parameters for oxidative stress and the presence of pro-inflammatory biomarkers [89]. This observation implies that inhalation of PM2.5 may initiate, or predispose individuals to tissue remodeling in the healthy lung. There is also evidence that exposure to PM2.5 during embryogenesis predisposes the embryonal lungs to develop chronic inflammatory lung diseases later in life [90]. The mechanism by which PM2.5 activates tissue remodeling is unclear, it may involve immune responses or may be independent from the immune system; the available literature does not provide sufficient data for a clear picture.

Inhaled PM2.5 was deposited in the mucosa, where it caused epithelial cell damage, stimulated the release of inflammatory mediators, and increased the risk of allergic response [90,91,92,93]. PM2.5 reduced the flexibility of alveolar epithelial cells by activating TGF-β [91,92]. Mass spectrometry analysis of the metabolome in bronchial epithelial cells showed that PM2.5 reprogramed epithelial cell function. This reprogramming was most likely controlled through epigenetic modifications of the metabolism necessary to provide energy, which suggest the involvement of mitochondria [94]. PM2.5 stimulated similar signaling signatures as reported for IgE (immunologic) and allergens, and thus will lead to the same end-effect on airway wall remodeling.

Regarding inflammation, the exposure of nasal epithelial cells to PM2.5 shifted the cytokine pattern from type-1 to type-2 in a rat model [93]. In rats, the inhalation of PM2.5 caused mitochondrial dysfunction in lung epithelial cells, which was characterized by increased mitochondrial radical oxygen species (ROS) production [94]. It thereby reduced mitochondrial oxygen consumption, decreased ATP production, and caused mitochondria fragmentation, especially the rupture of mitochondrial inner-membrane cristae in epithelial cells [94]. Air pollution damaged the mitochondrial electron transport chain, thereby activating alveolar epithelial cell apoptosis through its contents of PM2.5 [95]. This effect of PM2.5 might explain the damaged epithelium, which was documented in chronic inflammatory airway diseases [96].

Air pollution contains particles that are smaller than PM2.5, which may be even more damaging to the lung tissues, and this might include immunologic response of tissue forming and immune cells. It has been reported that PM0.1 not only modified the function of the epithelial cells, it even accumulated inside the mitochondria of alveolar epithelial cells, where it activated ROS production and thereby induced apoptosis [97]. These studies indicated that the chemical structure of the fine dust particles may be less important than the size, and thereby the penetration into the lung. It is not known how particles activate cells, but we know from the past that the inhalation of dust in the mining industry caused lasting damage to the lung. It has to be considered that fine dust may activate similar mechanisms as coal or silicon dust [98], and thereby activates remodeling, as well as the immune system in the lung.

The effect of PM2.5 on lung epithelial cells may be linked to pattern recognition proteins, especially to Toll-like receptors (TLRs), which also are important mediators of stress protein response. In animal models, the exposure to PM2.5 stimulated inflammation through TLR2/TLR4 and MyD88 signaling [99,100]. In human epithelial cells, the exposure to PM2.5 from indoor dust activated autophagy through activating TLR4 and Nuclear Factor kappa-B (NFκB) [101]. Interestingly, TLR4 was assumed to mediate the inflammatory and remodeling stimulating effect of HSP60 in vascular smooth muscle cells [102]. The latter has been released by epithelial cells of asthma patients and triggered fibroblast remodeling and inflammation through increasing mitochondria number and activity [79].

Regarding epigenetic predisposition to asthma, air pollution has been suspected to modify gene expression during childhood, but the mechanism remains elusive [103,104]. Importantly, exposure to PM2.5 during pregnancy induced mitochondrial DNA strand breaks and methylation, which persisted through childhood and may be responsible for chronic airway diseases later in life [105]. The causative role of PM2.5 exposure early in life and later development of chronic inflammation of the lung is supported by an animal model. This study showed that exposure to PM2.5 early in life induced epigenetic predisposition to asthma-like symptoms such as increased oxidative stress and extracellular matrix remodeling, which were inherited to the next generations [106]. Asthma and allergic diseases can be inherited, but there was no gene or gene pattern identified that is responsible for this phenomenon [107]. It is likely that the interaction between environmental factors such as allergens, dust, or even stress, can activate epigenetic events, but how these are becoming persistent and even inheritable remains to be investigated.

Interestingly, Rhinovirus infection in children who later developed asthma (*n* = 45) also caused DNA-methylation in specific regions that were associated with asthma [108]. In epithelial cells, the infection with respiratory syncytial virus induced the methylation of histones H3 and the action of lysine methyl-transferase G9a both modified the expression of interferon-γ [109]. In regards to epigenetic events, in non-smokers, PM2.5 caused the same methylation pattern of the gene encoding aryl-hydrocarbon receptor repressor, as observed earlier in cigarette smokers [110]. Similarly, PM2.5 caused DNA hypo-methylation and histone hyper-methylation randomly, thereby modifying the accessibility of genes for transcription proteins [111]. In the context of asthma, methylation of histone H3 is mainly catalyzed by PRMT1, which is a novel mediator of airway remodeling in asthma [79,112,113,114]. The expression of PRMT1 was cell type specific and responded to several well-known asthma triggers including TGF-β1 and IL-4 [112,115]. In human bronchial sub-epithelial fibroblasts, TGF-β1 regulated PRMT1 expression via Smad2/3 and C/EBP-β [114]. This might explain the loss of microRNA-19a and the subsequently deregulated constitutive expression of PRMT1 in mesenchymal airway wall cells of patients with moderate to severe asthma [113].

Only a few studies have been reported on the effect of PM2.5 on smooth muscle cells, mainly focusing on vascular remodeling. PM2.5 stimulated the expression of fibrogenic mediators in human airway smooth muscle cells, which might be linked to increased sub-epithelial fibrosis in asthma [116]. Air borne PM2.5 induced the calcification of blood vessels through NFκB and p38 MAPK, thereby increasing smooth muscle cell proliferation [117,118]. As mentioned earlier, the activation of NFκB by PM2.5 might be mediated through TLR4 [101]. In human airway smooth muscle cells, exposure to PM2.5 increased cell migration and therefore may contribute to the increased mass of smooth muscle cells in asthma [119].

Neutrophils and IL-17 were indicated to contribute to inflammation and remodeling in non-allergic asthma, caused by either infections, or chemicals, or other environmental factors. In regards to micro-particles contained in diesel-exhaust PM2.5 it was suggested that these can induce the expression of IL-17, and thereby stimulate inflammation and airway wall remodeling through neutrophils [120]. In an asthma model Il-17 activated neutrophils to secret neutrophil elastase, that modified the micro-environment of airway smooth muscle cells, leading to remodeling [121]. This effect might be linked to the earlier discussed stimulation of remodeling by the modification of the extracellular matrix composition, but the direct proof that this is a mechanism contributing to remodeling in asthma is missing.

**Figure 4:**
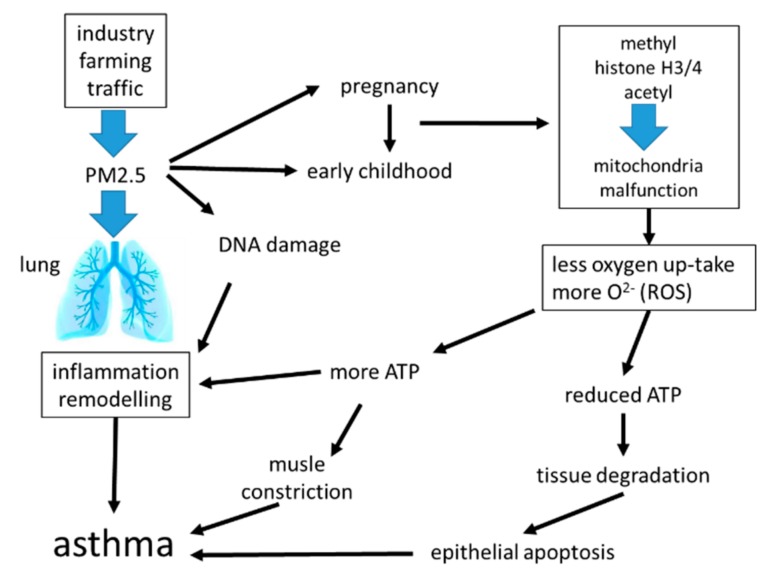
How does inhaled particulate matter size < 2.5 micrometers (PM2.5) predispose the lung during late embryogenesis and early childhood to develop chronic inflammatory lung diseases later in life? A major contributor to cell malfunction seems to be the PM2.5 induced dysfunction of mitochondria, which can be linked to many of the well-documented changes of the airway tissue structure and function that characterize chronic inflammatory lung diseases and airway wall remodeling.

A summary of the known effects of PM2.5 on inflammation and airway remodeling is shown in Figure 4.

## 6. Mechanisms that Control Remodeling in Non-Allergic Asthma

Non-allergic asthma can result from physical or psychological stress, as well as from changes in humidity or temperature. The mechanism(s) by which non-allergic asthma is triggered by the above described environmental factors or conditions is uncertain. It can be speculated that the release of stress proteins such as HSPs from epithelial cells may play a role, as has recently been reported in regard to bronchial thermoplasty therapy [79].

In this context, it is interesting that asthma exacerbation was linked to the presence of different HSPs produced by microorganisms, especially of HSP60 of *Chlamydia pneumoniae* [122,123]. The production of *C. pneumoniae* HSP60 might explain the ligand independent activation of the glucocorticoid receptor in human airway smooth muscle cells [124]. Furthermore, intracellular HSPs function as chaperones that control the interaction of the glucocorticoid receptor with steroids and its translocation into the nucleus [125]. Thus, a deregulated expression of HSPs might also explain steroid resistance in asthma patients. Other micro-organisms might affect airway smooth muscle cell remodeling by similar mechanisms [62,64,65,66], but more details need to be investigated.

Drug-induced asthma was linked to increased HSP70 secretion [126], while others reported that HSP70 counteracted formaldehyde-induced epithelial cell death [127]. The anti-inflammatory effect of exogenous HSP70 was supported in a mouse model for airway inflammation [128]. Exogenous HSP90 may induce epigenetic switches leading to asthma [129]. Recently it was suggested that HSP90 mediated viral infections and house dust mite-induced loss of epithelial barrier function [130,131,132,133,134]. However, the role of HSPs in the pathogenesis of asthma or other chronic inflammatory lung diseases is not well studied and has to be investigated in more detail. Importantly, the effects of exogenous HSPs should not be confused with those of intracellular HSPs. The role of the latter is much better studied and in general intracellular HSPs function as chaperons, which protect the cells from stress [135].

Changes in the hormone status during adolescence or as the result of malnutrition can also cause asthma. Adult onset asthma may be linked to steroid resistance, which seems to be age dependent in asthma and chronic obstructive pulmonary disease (COPD) [136,137]. The link to the hormone status affects mainly adult onset asthma in females after the menopause [138]. However, this link between gender and adult onset asthma was recently challenged in a Mexican cohort consisting of 403 participants [139].

Obesity is another unexplainable risk factor for asthma, which is especially important during childhood, with reduced physical activity [140]. Obesity as a risk factor for asthma has consequences for therapy and patients should be treated cautiously with steroids, which are known to increase accumulation of body fat [141]. The molecular mechanism linking obesity to asthma is not clear and has been linked to insulin resistance. Other factors that may link obesity to asthma include intestinal microbiota, and the metabolism of fatty acids [142].

## 7. Early in Life Epigenetic events and the Predisposition of the Lung to Develop Asthma

**Figure 5:**
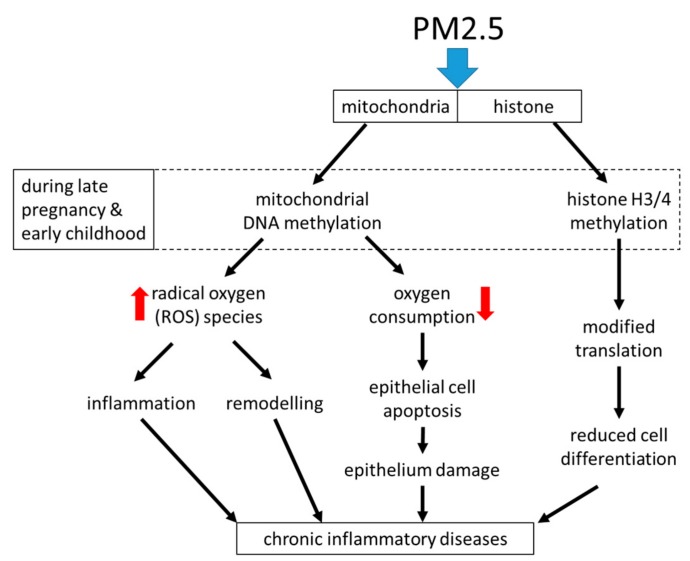
The epigenetic effects of PM2.5 and their consequences for airway wall remodeling and inflammation in asthma. Exposure of cells to PM2.5 activates epigenetic mechanisms including mitochondria mass/activity and histone modification by methylation. Mitochondria activity increases the production of oxygen radicals (ROS) and lowers the oxygen consumption, which lead to inflammation and remodeling in a cell type specific pattern. Protein methylation affects gene accessibility by transcription factors and also affects protein function. This all results in inflammation and remodeling.

Epigenetic events that are initiated early in life present a novel hypothesis for the origin of chronic inflammatory lung diseases (Figure 5). Especially, epigenetic events during the third trimester of pregnancy and the first 6 years of childhood condition the lung to develop chronic inflammatory lung diseases (asthma, COPD) later in life [104,143,144,145]. Lung tissue specific histone acetylation, mitochondria activity, and disease specific microRNA expression profiles have been reported in asthma patients and were linked to both the immune response and airway wall remodeling [146,147]. Interestingly, these epigenetic events have been reported as being inheritable over three generations by an unknown mechanism [104,107,145,148].

DNA hyper-methylation is one of the most investigated epigenetic events linked to childhood asthma. Most studies reported a hyper-methylation of CG-region [149], while others showed a hypo-methylation of the same sites in children with asthma [150]. Assessing the DNA methylation of 1629 children, Wu et al. reported hyper-methylation of IL-10 and LIM Domain Only-2 (LMO2) [150]. Interestingly, there is evidence that childhood asthma, as well as COPD (later in life), is linked to a modification of the TGF-β signaling pathway by epigenetic events earlier in life [151]. Hyper-methylation of IL-4, IL-5, and eosinophil peroxidase were reported in two cohorts of children with asthma and were linked to impaired IP3-mTOR signaling [152]. The latter finding could be linked to the effect of IgE on downregulating the Akt inhibitor PTEN [51]. However, there is little consistency comparing the results of methylation patterns from all these studies as the cause of asthma.

Summarizing the above (Figure 5), airway wall remodeling in asthma can neither be attributed to immunological nor non-immunological triggers. It is indicative that further investigations have to determine whether specific asthma sub-types are linked to specific structural changes of the airways. Provocatively, one could suggest that specific structural changes in the asthmatic airways may be the key to classify asthma pheno- and endo-types. However, the permission to perform such studies will be difficult, as they would require bronchial tissue samples from different bronchi in a large number of asthma patients with clearly defined clinical symptoms, severities, and age groups.

## Abbreviations

Akt	activated protein kinase B
ATP	adenosin-tri-phosphate
bFGF	basic fibroblast growth factor
COPD	chronic obstructive pulmonary disease
CD	cluster of differentiation
Erk	extracellular signal-regulated kinase
HSP	heat shock protein
IgE	immune-globulin E
IL	interleukin
IP3K	Inositol-trisphosphate 3-kinase
MAPK	mitogen activated protein kinase
miR	microRNA
mTOR	mammalian target of rapamycin
p70S6K	ribosomal protein S6 kinase
PGC	peroxisome proliferator-activated receptor gamma coactivator
PGE	prostaglandin E
PM	particulate matter
PPAR	peroxisome proliferator-activated receptor
PRMT	protein arginine methyltransferase
PTEN	phosphatase and tensin homolog
STAT	signal transducers and activators of transcription
TGF	transforming growth factor
Th	T-helper cell
TSLP	thymic stromal lymphopoietin
TLR	toll-like receptor
WHO	world health organization
Wnt	wingless-related integration site
